# L-T4 Therapy in the Presence of Pharmacological Interferents

**DOI:** 10.3389/fendo.2020.607446

**Published:** 2020-12-22

**Authors:** Salvatore Benvenga

**Affiliations:** ^1^Department of Clinical and Experimental Medicine, University of Messina, Messina, Italy; ^2^Master Program on Childhood, Adolescent and Women’s Endocrine Health, University of Messina, Messina, Italy; ^3^Interdepartmental Program of Molecular & Clinical Endocrinology and Women’s Endocrine Health, University Hospital, A.O.U. Policlinico G. Martino, Messina, Italy

**Keywords:** levothyroxine, hypothyroidism, pharmacological interferents, metabolic complications, cardiovascular complications

## Abstract

Pharmacological interference on L-thyroxine (L-T4) therapy can be exerted at several levels, namely from the hypothalamus/pituitary through the intestine, where the absorption of exogenous L-T4 takes place. A number of medications interfere with L-T4 therapy, some of them also being the cause of hypothyroidism. The clinician should be aware that some medications simply affect thyroid function tests with no need of modifying the dose of L-T4 that the patient was taking prior to their prescription. Usually, the topic of pharmacological interference on L-T4 therapy addresses the patient with primary hypothyroidism, in whom periodic measurement of serum thyrotropin (TSH) is the biochemical target. However, this minireview also addresses the patient with central hypothyroidism, in whom the biochemical target is serum free thyroxine (FT4). This minireview also addresses two additional topics. One is the costs associated with frequent monitoring of the biochemical target when L-T4 is taken simultaneously with the interfering drug. The second topic is the issue of metabolic/cardiovascular complications associated with undertreated hypothyroidism.

## Introduction

Prevalence of undiagnosed primary hypothyroidism (PH) in Europe is 4.76% (95% CI 2.98–6.79%), precisely 4.11% (3.05–5.31%) for subclinical hypothyroidism (SCH) and 0.65% (0.38–0.99%) for overt hypothyroidism (OH) (1), paralleling the American prevalence of 4.67, 4.34, and 0.3%, respectively (2). PH prevails in females, and in those aged ≥65 years (1). Central hypothyroidism (CH) was considered 1,000-fold rarer than PH, but it may be just 20-fold rarer (3). L-thyroxine (L-T4) monotherapy is the standard treatment for PH (4–6) and CH (4, 5, 7), and SCH patients candidate for replacement (4, 5, 8).

Thus, L-T4 is one top prescribed medication (9). However, ≈20% of patients with PH (10–13) and ≈20% (14) or 40% (15) of patients with CH are undertreated, co-administration of interfering medications (IM) being one major cause. Of 2,292 patients with PH (12), 42.7% had abnormal serum TSH (28.3% undertreated and 14.4% overtreated). Strikingly, only 52.1% of patients were told not to take L-T4 along with IM (12).

As mentioned below, there may be consequences on health from undertreated hypothyroidism (UTH).

## The Pharmacological Interference on L-T4 Therapy

Such interference was covered by reviews (11, 16–19) chapters of books (10, 20–22), and guidelines (4, 5) with pertinent recommendations (4, 5) shown in **Table 1**.

**Table 1:** Recommendations concerning medications that interfere with L-T4 therapy in the latest American Thyroid Association Guidelines on hypothyroidism*.

Year (ref.)	Question and Recommendation
**2012 (**4**)**	
	How should patients with hypothyroidism be treated and monitored?26. In patients receiving L-thyroxine treatment for hypothyroidism, serum TSH should be remeasured within 4–8 weeks of initiation of treatment with **drugs that decrease the bioavailability or alter the metabolic disposition of the L-thyroxine dose**.Grade A, BEL 1
	When should endocrinologists be involved in the care of patients with hypothyroidism?28. Physicians who are not endocrinologists, but who are familiar with the diagnosis and treatment of hypothyroidism *should be able* to care for most patients with primary hypothyroidism. However, patients with hypothyroidism who fall into the following categories should be seen in consultation with an endocrinologist. These categories are (i) children and infants, […] and (ix) unusual **causes of hypothyroidism such as those induced by agents that interfere with absorption of L-thyroxine, impact thyroid gland hormone production or secretion, affect the hypothalamic–pituitary–thyroid axis (directly or indirectly), increase clearance, or peripherally impact metabolism**.Grade C, BEL 3
**2014 (**5**)**	
	Are there medications and supplements that should not be co-administered with levothyroxine in order to avoid impaired absorption?3b. We recommend that where feasible, levothyroxine should be separated from other potentially interfering medications and supplements (*e.g.*, **calcium carbonate** and **ferrous sulfate**). A 4-h separation is traditional but untested. Other medications (*e.g.*, **aluminum hydroxide** and **sucralfate**) may have similar effects but have been insufficiently studied.Weak recommendation. Weak quality evidence.
	What medications may alter a patient’s levothyroxine requirement by affecting either metabolism or binding to transport proteins?3e. Initiation or discontinuation of **estrogen** and **androgens** should be followed by reassessment of serum thyrotropin at steady state, since such medications **may alter the levothyroxine requirement**. Serum thyrotropin should also be reassessed in patients who are started on agents such as **tyrosine kinase inhibitors** that affect thyroxine metabolism and thyroxine or triiodothyronine deiodination. Serum thyrotropin monitoring is also advisable when medications such as **phenobarbital, phenytoin, carbamazepine, rifampin, and sertraline** are started.Strong recommendation. Low-quality evidence.

*Bold-face print is by the author of this minireview, in order to highlight terms and/or parts of interest.

**Table 2** summarizes the mechanism(s) of interference and suggestions. The next paragraphs illustrate the author’s experience. Of 210 L-T4-treated adult patients (tablet formulations) who were referred to SB in one year, 27 (13%) had UTH (11). In 8/27 (30%; 4% of 210), the explanation was pharmacological, all eight taking ≥1 drug that causes L-T4 malabsorption (proton-pump inhibitors [PPIs], calcium carbonate, ferrous sulfate) (11). A study with practitioners enrolled L-T4-treated PH (tablet formulations) that, for ≥2 years was not associated with drugs causing L-T4 malabsorption and that, for another ≥2 years, it was (23). Of 10,496 persons, 730 (7.0%) had L-T4-treated PH, of whom 391 (5.4%; 3.7% of 10,496) were taking those IM, with 114 enrolled (age 65.6 ± 12.7 years). PPIs were the leading IM (95/114 [83%]), either alone (n = 71 [62%]) or associated with other IM (n = 24 [21%]), followed by calcium salts (18/114 [16%]).

**Table 2:** Medications that may interfer on with L-T4 therapy and suggestions for management in addition to monitoring of thyroid function.

Medication	Mechanism(s)	What to do
Antacids, nonabsorbable^§^	Physical interaction with T4	Concomitant use to be avoided (intake separated by ≥4 h)
Calcium salts^§^	Physical interaction with T4	Concomitant use to be avoided (intake separated by ≥6 h)
Cholestyramine^§^, Colesevelam^§^	Physical interaction with T4	Concomitant use to be avoided (intake separated by ≥4 h)
Phosphate binders§ and potassium binders	Physical interaction with T4	Concomitant use to be avoided (intake separated by ≥4 h)
Ferrous salts^§^	Physical interaction with T4	Concomitant use to be avoided (intake separated by ≥4 h)
Orlistat^§^	Physical interaction with T4?	Thyroid function to be monitored.L-T4 dose may need to be increased
Antacids, absorbable^§^	Increase of intragastric pH	Thyroid function to be monitored.L-T4 dose may need to be increased
Androgens/Anabolic steroids*	↓ both T4 binding to plasma proteins and TSH	Thyroid function to be monitored.L-T4 dose may need to be lowered
Estrogens*	↑ both T4 binding to plasma proteins and TSH	Thyroid function to be monitored.L-T4 dose may need to be increased
Tamoxifen*	↑ T4 binding to plasma proteins	Thyroid function to be monitored.L-T4 dose may need to be increased
Raloxifene *^§^	↑ T4 binding to plasma proteinsT4 malabsorption	Concomitant use to be avoided (intake separated by ≥12 h)L-T4 dose may need to be increased
Clofibrate*	↑ T4 binding to plasma proteins	Thyroid function to be monitored.L-T4 dose may need to be increased
Fluorouracil* and its prodrug capecitabine*	↑ T4 binding to plasma proteins	Thyroid function to be monitored.L-T4 dose may need to be increased
Corticosteroids*	↓ TSH	L-T4 dose may need to be lowered
Growth hormone	↑ T4 metabolism	Thyroid function to be monitored.L-T4 dose may need to be increased
Antiepileptics (carbamazepine*, phenytoin*, phenobarbital*, valproate, etc…)	↑ T4 metabolism; ↓ T4 binding to plasma proteins and ↓ TSH (phenytoin)	Thyroid function to be monitored.L-T4 dose may need to be increased
Lithium^†^	↓ T4 synthesis; ↓ T4 metabolism, ↑ TSH	Thyroid function to be monitored.L-T4 dose may need to be increased
Tricyclic antidepressants; SSRIs*	↓ TSH; SSRIs ↑ TSH	Thyroid function to be monitored.L-T4 dose may need to be increased
Rifampin*	↑ T4 metabolism	Thyroid function to be monitored.L-T4 dose may need to be increased
Metformin	↓ TSH	Thyroid function to be monitored.
Sulphonamides	↓ T4 synthesis	Thyroid function to be monitored.
Aminoglutethimide^†^	↓ T4 synthesis	Thyroid function to be monitored.L-T4 dose may need to be increased
Mitotane*	↑ T4 binding to plasma proteins. ↑ T4 metabolism, ↓ TSH	Thyroid function to be monitored.L-T4 dose may need to be increased
Bexarotene^†^	↑ T4 metabolism, ↓ TSH	Thyroid function to be monitored.L-T4 dose may need to be increased
Dopamine (≥0.4 mcg/kg/min), dopamine agonists	Transiently ↓ TSH	Change in L-T4 dose unnecessary
Octreotide (≥100 mcg/day), somatostatin analogs	Transiently ↓ TSH	Thyroid function to be monitored.L-T4 dose may need to be increased
Antiretroviral drugs	↑ T4 metabolism	Thyroid function to be monitored.L-T4 dose may need to be increased
Ethionamide^†^, para-aminosalicylic acid^†^	↓ iodine organification; thyroiditis	Thyroid function to be monitored.
Thalidomide, lenalidomide, pomalidomide	Thyroiditis	Thyroid function to be monitored.
Tyrosine kinase inhibitors*^†^	Thyroiditis; ↑ T4 metabolism; inhibition T4 and T3 cell transporters.	Thyroid function to be monitored.L-T4 dose may need to be increased
Cytokines, Interferons^†^	Thyroiditis	Thyroid function to be monitored.
Monoclonal anti CD52 (alemtuzumab)	Thyroiditis	Thyroid function to be monitored.
Monoclonal anti-CTLA-4 Ab (ipilimumab, tremelimumab)	Hypophysitis; thyroiditis	Thyroid function to be monitored.
Monoclonal anti-PD-1 Ab (nivolumab, pembrolizumab)	Thyroiditis; hypophysitis	Thyroid function to be monitored.
Furosemide	↓ T4 binding to plasma proteins	Change in L-T4 dose unnecessary
Heparin	↓ T4 binding to plasma proteins	Change in L-T4 dose unnecessary
Nicotinic acid	↓ T4 binding to plasma proteins	Change in L-T4 dose unnecessary
Salicylates (>2 g/day) and other nonsteroidal anti-inflammatory drugs	↓ T4 binding to plasma proteins	Change in L-T4 dose unnecessary

Ab, antibody/antibodies; CLTA-4, cytotoxic T lymphocyte-associated antigen 4; PD-1, programmed cell death-1 receptor; SSRIs, selective serotonin reuptake inhibitors.

Symbols: ↑, increased; ↓, decreased. Based on Jonklaas (10), drugs that “may alter the levothyroxine dose required by a patient” are indicated by one asterisk (*) if the mechanism is “by affecting T4 metabolism or transport”, and by the section sign (§) if the mechanism is “by affecting levothyroxine absorption”. The dagger (†) identifies medications that may trigger hypothyroidism, which is of the central type in the case of bexarotene, and may also be of the central type in the case of medications that act by lowering TSH secretion and causing hypophysitis.

In the literature, interfering medications are categorized variably, depending on mechanism, with some medications having multiple mechanisms. For instance, the 2012 American Thyroid Association (ATA) guidelines (4) considered four mechanistic categories, with information provided when mechanisms are multiple, namely: (i) direct and indirect effects on the hypothalamic–pituitary–thyroid axis; (ii) thyroid gland hormone production and secretion; (iii) increased clearance; (iv) interference with absorption. In the 2014 ATA guidelines (5), categories were two, viz. (i) impaired absorption, and (ii) altered metabolism or binding to transport proteins, with emphasis on the first category. In a book (20) medications are listed under five categories: (i) central TSH inhibition; (ii) absorption; (iii) synthesis and secretion; (iv) transport; (v) metabolism. In another chapter of this book (10), emphasis is given to drugs that may alter the L-T4 dose by affecting (i) T4 metabolism or transport, and (ii) L-T4 absorption. In another book (22), drugs are listed under two major categories, viz. interfering with (i) hypothalamic–pituitary–thyroid function, (ii) thyroid function.

Whenever “thyroiditis” appears in the second column of the Table (Mechanism), the clinical implication is that such silent thyroiditis may translate into monophasic thyrotoxicosis, monophasic hypohyroidism, or biphasic dysfunction (thyrotoxicosis followed by hypothyroidism). However, hypothyroidism may sometimes be permanent. In the case of lithium, a few cases of thyrotoxicosis have been reported. Interferon alpha, alemtuzumab, ipilimumab, tremelimumab, nivolumab and pembrolizumab may even trigger true Graves’ disease. Clearly, whenever a L-T4-treated hypothyroid patient experiences increased discharge of thyroid hormones (thyrotoxicosis or hyperthyroidism), because he/she is simultaneously taking a medication that causes such side-effect, L-T4 therapy has to be withdrawn. Upon rechecking thyroid function tests during the follow-up period in such patient, L-T4 replacement is started again.

Another study evaluated changes in number of L-T4 prescriptions and dose of L-T4 before and during exposure to potential drug–drug interactions (DDIs) (24). In 5,426 L-T4 users aged ≥18 years (7.5% of persons under care), prescriptions and doses of L-T4 increased during exposure by 6 and 5%, respectively, suggesting that clinicians increase the number of L-T4 prescriptions to achieve TSH levels as low as those before DDIs (24).

A retrospective study evaluated TSH changes in 10,999 Scottish residents who were prescribed L-T4 before starting IM (25). Iron, calcium, PPI, and estrogens increased TSH significantly, with an increase >5 mU/L in 7.5, 4.4, 5.6, and 4.3% patients, respectively. TSH decreased significantly (0.17 mU/L) in patients on statins and changed insignificantly in patients on H2 receptor antagonists or glucocorticoids (25).

Before continuing, SB informs that this minireview disregards the endocrine disruptors, and does not emphasize clinically inconsequential IM. Such medications are those listed at the end of **Table 2** (furosemide, heparin, salycylates, and other nonsteroidal anti-inflammatory drugs), which inhibit T4 binding to plasma proteins thus increasing serum FT4, and the unlisted propranolol (20). Also, a number of articles are dedicated to specific IM (26–40).

## Thyroid Dysfunction and Suboptimal Treatment Caused by Interfering Medications Occur in a Proportion of Patients

The Scottish study (25) is important because it reminds that not all L-T4-replaced hypothyroid patients taking IM become undertreated. For instance, a 3-month supplementation of 1,200 mg/d calcium carbonate reversibly increased TSH in 13/20 patients (65%), with TSH >4.0 mU/L in four (20%) (41). Overall, TSH increased from 1.60 ± 0.22 to 2.71 ± 0.43 mU/L (+69%). Calcium carbonate adsorbed T4 dose-dependently at pH 2 but not pH 7.4, thus explaining L-T4 malabsorption (41), and reduced T4 pharmacokinetics (42). Lower L-T4 bioavailability applies to other calcium salts (43). Those data (41) match data of SB (44). Fifty postmenopausal women with L-T4-treated PH started taking 600–1000 mg/d calcium carbonate ≤2 h after L-T4. UTH (TSH >4.12 mU/L) occurred in 9/50 women (18%). Overall, TSH increased from 1.93 ± 0.51 mU/L to 3.33 ± 1.93 mU/L (+73%), but when all women took calcium 6–8 h after L-T4, all had TSH <4.12 mU/L (2.16 ± 0.54 mU/L) (44).

Confirming previous data (45), in 37 PH patients under stable L-T4 replacement and in whom PPIs were administered subsequently, TSH increased by 28%, with seven patients (19%) having post-PPI TSH levels >5 mU/L (46). A mean increase of 20 μg/d L-T4 (+35%) was necessary (46).

In 71 L-T4-treated PH patients who started the antituberculosis drug rifampin, an increased L-T4 dose was required for 50% of 46 patients (TSH-suppressive group) and 26% of 25 patients (replacement group) (47). Lack of thyroid remnant, time interval between starting rifampin and TSH measurement, and baseline L-T4 dose/kg body weight were significant risks for UTH (47). Ethionamide and para-aminosalycilic acid (PAS) are used in multidrug-resistant tuberculosis (MDR-TB). After initial reports (48–50), a meta-analysis on 6,241 MDR-TB patients (31) showed that PH prevalence in MDR-TB patients averaged 17.0%, with ethionamide and PAS were the most frequently reported drugs associated with hypothyroidism. Tubercolosis is a common opportunistic infection in HIV-seropositive persons, and antiretroviral therapy (ART) may induce PH. Of 69 HIV-infected MDR-TB patients under anti-TB and antiretroviral therapies, 37 (54%) had PH (51). Co-administration of PAS and ethionamide doubled the risk of hypothyroidism (RR = 1.93) (51). In MDR-TB patients receiving anti-TB, one-fourth developed PH: 32% in patients who received a regimen containing ethionamide, 35% in patients who received a regimen containing PAS, and 44% in HIV-positive patients on ART (52).

Immune reconstitution therapy (IRT)-induced either autoimmune hyperthyroidism with/without ophthalmopathy or autoimmune hypothyroidism may occur following: (i) highly active antiretroviral therapy (HAART) of HIV infection, (ii) alemtuzumab treatment for active relapsing-remitting multiple sclerosis, and (iii) allogeneic bone marrow transplantation or hematopoietic stem cell transplantation (30). No recommendation for managing IRT-related thyropathies addressed possible changes in L-T4 doses (30). Hypothyroid HAART-treated patients needing increased L-T4 doses were reported (53–55). HAART drugs were ritonavir (48), abacavir–lamivudine and lopinavir–ritonavir (54), and lopinavir/ritonavir plus zidovudine and lamivudine (55). Switch from lopinavir/ritonavir to nelfinavir failed to achieve euthyroidism (55), which was restored when dolutegravir was substituted for lopinavir–ritonavir (54). However, 826 well-treated HIV-infected persons, 95% of whom with undetectable viral replication, did not differ from 2,503 uninfected controls for prevalence of hyperthyroidism (0.8 *vs.* 0.8%) or hypothyroidism (3.8 *vs.* 4.6%) (56).

Hypothyroidism occurs in around 20 or 5–10% of thalidomide- or lenalomide-treated patients (57, 58). Of 170 patients under lenalidomide, 6% had thyroid dysfunction (hypothyroidism > thyrotoxicosis), which prevailed in patients with known *vs.* unknown thyroid dysfunction (17 *vs* 6%) (58). Unfortunately, the article fails to specify how many patients were under L-T4 and how many needed increased L-T4 doses (58). With just two cases reported (59, 60), the rate of pomalidomide-induced hypothyroidism is unknown. Interestingly, thyroid hormone autoantibodies appeared in 14% of thalidomide or lenalidomide-treated patients (61).

Autoimmune thyroiditis is also caused by monoclonal Ab to programmed cell death-1 receptor (nivolumab and pembrolizumab), and Ab to cytotoxic T lymphocyte-associated antigen-4 (ipilimumab, tremelimumab) (27, 37–39). Rates of hypothyroidism are 0–40% (nivolumab), 0–11.5% (pembrolizumab), 0–9% (ipilimumab), and 0–5% (tremelimumab) (38).

Tyrosine kinase inhibitors (TKI) may cause *de novo* hyperthyroidism or hypothyroidism, or worsen pre-existing hypothyroidism and increase L-T4 requirements (27–29). Upgraded titration of L-T4 was reported in athyreotic patients prescribed imatinib, motesanib, sorafenib, sunitinib, and vandetanib (5, 62, 63). Nilotinib and dasatanib rarely alter L-T4 requirements (27). One mechanism for the L-T4 increased requirements, further to T4 increased metabolism, can be inhibition of MCT8-dependent T3 and T4 uptake (64), since MCT8 is expressed by the gastrointestinal tract (65).

Other anti-cancer IM are mitotane (see below) and aminogluthetimide, a first-generation aromatase inhibitor used for advanced breast cancer (ABC), but whose side-effects and need for concomitant hydrocortisone limited its utility (66). Of 29 aminoglutethimide-treated patients with prostate cancer, nine (31%) developed hypothyroidism (TSH >10 mU/L) (67). Of 32 women under aminoglutethimide + hydrocortisone for ABC, 17 (53%) had hyperthyrotropinemia before treatment. In 15/17 patients, TSH increased further, with seven developing OH (68). Because aminoglutethimide blocks adrenal steroidogenesis, it is used to treat Cushing’s syndrome (69).

OH or SCH develop in up to 15 or 34% of lithum-treated patients (20), the annual rate of developing PH being 1.5%, (6.4% in thyroid antibody-positive and 0.8% in thyroid antibody-negative individuals) (70). Women <60-year-old are at greatest risk to develop thyroid disease (71). Indeed, women develop OH or SCH three times more frequently than men (25.8 *vs.* 8.7%), with prevalence among women exceeding 50% by the age of 65 years (35). During tricyclic antidepressant therapy, TSH levels are unchanged, and T4 and FT4 levels decrease within the euthyroid range, though other studies reported unchanged thyroid function tests (TFTs) (72). Selective serotonin reuptake inhibitors (SSRIs) affect TFTs variably, usually with no or downward changes of FT4 and FT3, and no or upward change of TSH within the corresponding reference range (72). L-T4 requirements increased in nine L-T4-treated hypothyroid patients under sertraline (73). Confirming the sertraline-T4 interaction, in two patients under TSH-suppressive L-T4 therapy, TSH levels rose into the normal range (73). A 3-month duration study was conducted in 57 patients with major depression and 10 control patients (72). The study patients were hypothyroid on adequate L-T4 therapy (n = 28) who were randomized to fluoxetine (n = 13) or sertraline (n = 15), and euthyroid (n = 29) who were treated with fluoxetine (n = 15) or sertraline (n = 14). Controls had hypothyroidism on adequate L-T4 therapy without depression (72). No changes occurred in the L-T4-replaced hypothyroids under either SSRI. In response to a letter (74) commenting that difference with the early study (73) could be that” *many … patients … were athyreotic*”, the authors admited that “*in most [patients] the cause of hypothyroidism was autoimmune*” (75). Noteworthy, a 41-year-old man with a history of bipolar disorder and schizophrenia had myxedema coma after therapy with sertraline and ariprazole (76). After discharge, TSH remained high (34 IU/ml) on sertraline, ariprazole and 200 µg/d L-T4.

SCH may occur reversibly following administration of antiepileptic drugs (AED), particularly carbamazepine, phenytoin, valproate (77), oxcarbazepine, but not lamotrigine, levitracetam, tiagabine and vigabatrine (36). AED administration in L-T4-replaced patients with either PH (78) or CH (32) requires higher L-T4 doses.

The anti-obesity drug orlistat inhibits gastro-intestinal lipases and is minimally absorbed. Based on data of the UK Medicines Information pharmacists (79), only two cases of interaction between orlistat and L-T4 were reported (80, 81), most likely *via* T4 malabsorption. It was recommended that L-T4 and orlistat *“should be separated by 4 hours, and increased monitoring of […] thyroid hormone levels may be prudent*” (79).

Through acceleration of both thyroid hormone and cortisol metabolism, initiation of growth hormone (GH) replacement may unmask CH and hypoadrenalism (82–84). Also, patients already under L-T4 replacement need increased L-T4 doses (82, 84). Unmasking of CH and increased L-T4 doses occurred in 30/84 (36%) and 25/159 (16%) patients, respectively (84).

## Pharmacological Interference at Central Level

The antipsycotic dopamine antagonists (*e.g.*, phenothiazines, haloperidol, domperidone, metoclopramide, cimetidine), antidepressive tricyclics and *α*-methyldopa slightly increase serum TSH without altering thyroid function (17). Though dopamine and its agonists (bromocriptine, cabergoline), somatostatin analogs (octreotide), dobutamine, amphetamines and corticosteroids inhibit TSH, patients taking chronically these medications have no sustained decreases of TFTs. Consequently, CH does not develop (34, 85). Reversible inhibition of TSH secretion is also observed with AED, particularly phenytoin (20).

Anti-CTLA-4 Ab and anti-PD-1 Ab induce autoimmune hypophysitis (27, 37–39), with rates of 0–17.4% (ipilimumab), 0–2.6% (tremelimumab), 0–0.9% (nivolumab), and 0–1.2% (pembrolizumab) (38). In anti-CTLA4-Ab-induced hypophysitis, ACTH- and TSH-deficiency are frequent (38).

Metformin lowers serum TSH in L-T4-treated OH and L-T4-untreated SCH patients, but not in euthyroid patients (33). After its chronic administration, FT4 was unchanged and TSH decreased in L-T4-treated or L-T4-untreated hypothyroid diabetics, but not in euthyroid subjects (86).

A single oral dose of clofibrate decreased significantly hyperthyrotropinemia of PH patients (87). Clofibrate did not change discernibly basal and TRH-induced TSH secretion in euthyroids. Similar results were given by meclofenoxate hydrochloride (87), suggesting that both drugs inhibit TSH secretion in PH patients possibly acting on the hypothalamus/pituitary (87). Clofibrate also increased serum T4-binding capacity of TBG, lowering serum FT4 in 11/12 hyperlipoproteinemic patients (92%) (88).

The adrenocytolytic drug mitotane is used to treat adrenocortical carcinoma (ACC) (89). In 17 patients with radically resected ACC, mitotane was administered associated with glucocorticoid replacement therapy (90). Excluding three patients under L-T4 treatment and one with SCH, during the first year, FT4 became subnormal in 12/13 evaluable patients (92%), with L-T4 replacement started after 9 months in four. At last follow-up, FT4 levels were unchanged compared with the 12 month-evaluation, but another three patients needed L-T4 replacement. Five women with mitotane-treated ACC showed features of CH, namely low FT4, normal FT3 and TSH, with impaired TSH response to TRH (91). Mitotane increased serum FT3/FT4 ratio, suggesting enhanced T4 to T3 conversion, a compensatory mechanism of hypothyroidism (91). CH was reported in a girl with mitotane-treated ACC (92); full restoration of FT4 required increasing the L-T4 dose. After completing chemotherapy, TFTs remained normal, and L-T4 replacement was discontinued.

The retinoid bexarotene causes CH in almost all patients (22), *via* TSH suppression (93) and non-deiodinase-mediated thyroid hormone increased metabolism (94).

## Hypothyroid Patients Hospitalized in Intensive Care Units (ICU)

Among 133 such patients, TFTs were not performed in 29 (21.8%), replacement therapy was not prescribed for >7 days in 23 (17.3%) and was omitted in three (2.2%) (95). Nine hypothyroid patients who stayed at ICU for 12–45 days received H2-blockers as routine prophylaxis against gastroduodenal bleeding and were nasogastrically fed a calorically dense solution (96). This solution was stopped 2 h before and resumed 2 h after L-T4 administration (crushed tablets) nasogastrically. After the first 4–8 days at ICU, with L-T4 doses unchanged, TSH of all patients increased significantly to 5.69 mU/L (IQR = 3.87–6.83) from 1.52 mU/L (IQR = 0.79–3.8) at admission. L-T4 dose was increased in 8/9 patients (88.9%) from 86.1 ± 41.6 to 125 ± 39.5 µg/d (54.4 ± 31.6%). Upon discharge, TSH returned to levels (2.8 mU/L [IQR = 1.4–5.5]) insignificantly different from baseline. The increased L-T4 requirements were attributed to increased gastric pH by H2-blockers (and subsequent decreased L-T4 absorption) and increased T4 to reverse-T3 shunting (due to enhanced type-III deiodinase activity during critical illnesses) (96). In 320 ICU patients, the most frequent clinically significant drug–enteral nutrition interactions concerned phenytoin, warfarin and L-T4 (97).

## Current Research Gaps

Concerning IM-induced UTH, few data are available on two issues (23, 44, 98, 99): (i) metabolic/cardiovascular complications, (ii) costs of frequent assays to monitor L-T4 therapy.

In the aforementioned study on 114 L-T4-treated PH patients (23), exposure to IM (32.1 ± 6.9 months) significantly increased TSH from 1.27 ± 1.34 to 2.81 ± 3.62 mU/L (+121%), and proportions of TSH >4.12 mU/L from 4.7 to 18.5% and >2.50 mU/L from 20.2 to 53.5% compared to the non-exposure period (35.4 ± 9.7 months). Some complications ensued: aggravation of pre-existing or *de novo* onset of any of metabolic syndrome (MS), impaired fasting glycemia (IFG), diabetes, dyslipidemia, hypertension, cardiovascular disease (CVD). Seventy-six patients (67%) had complications, whose rates of TSH >4.12 or >2.50 mU/L were significantly greater than in the 36 complication-free patients (22 *vs.* 11%, or 35 *vs.* 17%). TSH in patients with complications *vs.* complication-free patients were 118% greater during exposure (3.44 ± 4.08 *vs.* 1.58 ± 1.98 mU/L) (23). In the above study on 50 postmenopausal L-T4-treated PH women (44), TSH increased significantly from 1.93 ± 0.51 (before calcium; setting 1) to 3.33 ± 1.93 mU/L (calcium taken ≤2 h after L-T4; setting 2), and was significantly lower than 2.16 ± 0.54 mU/L (calcium taken 6–8 h after L-T4; setting 3). Total cholesterolemia (TC), fasting glycemia (FG), systolic and diastolic blood pressure (SBP and DBP) were also significantly higher in setting 2 *vs.* settings 1 and 3. For every 1.0 mU/L increase within the TSH range of 0.85–6.9 mU/L, TC, FG, SBP, and DBP increased by 12.1 mg/dl, 3.12 mg/dL, 2.31 mmHg, and 2.0 mmHg, respectively (44). As summarized elsewhere (100), our data (23, 44) agree with other data outside of the IM setting, which show that as TSH levels increase within its euthyroid range, metabolic and cardiovascular outcomes worsen progressively. For instance, comparing TSH of 3.0–3.5 mU/L with 0.50–0.99 mU/L, the OR for hypertension was 1.98 in men and 1.23 in women (101). Within the reference range of 0.50–3.5 mU/L, blood pressure increased linearly with increasing TSH (SBP by 2.0 mmHg in men and 1.8 mmHg in women, DBP increased by 1.6 and 1.1 mmHg) (101). In 12,584 adults with normal TSH levels, the frequency of diabetes, hypertension, and hypercholesterolemia, increased significantly across TSH tertiles (diabetes = 6.3, 7.7, and 9.1%; hypertension = 20.9, 24.8, and 29.8%; hypercholesterolemia = 13.2, 15.2, and 19.4%) (102). Moreover, SCH might favor diabetic complications with an OR of 1.74 (nephropathy), 1.42 (retinopathy), 1.85 (peripheral arterial disease), and 1.87 (peripheral neuropathy) (103).

High-normal TSH levels impact unfavorably on mortality. In 9,020 adults, SCH (TSH >5.60 mIU/L) and high-normal TSH (1.96–5.60 mIU/L) were associated with increased all-cause mortality (HR = 1.90 and 1.36) *vs.* the middle-normal TSH group (1.20–1.95 mIU/L) (104), with CVD mediating 14.3 and 5.9% of the association, respectively (104). A study on 611 hospitalized elderly patients evaluated all-cause mortality up to 66 months after discharge (105) and concluded that (i) in treated hypothyroid patients, median TSH levels of 5–10 IU/L associated with increased mortality, (ii) treatment should aim at achieving euthyroidism to improve survival (105).

Undertreated CH is associated with increased weight, BMI, larger waist circumference (106), and higher fat mass, worse lipid profile (total cholesterol, LDL-cholesterol, HDL-cholesterol, triglycerides) (107), with increased risk for cardiovascular morbidity (108).

Concerning costs, these were estimated by four simulations, based on 5% rate of PH (4.5% SCH, 0.5% OH), three rates (10, 50, and 80%) of L-T4-treated SCH patients, 440,000 to 3,608,000 UTH persons, and a total of 125,800 to 1,031,888 persons with calcium-related UTH and iron-related UTH (98). At euro 20 for one TSH test, the total cost for five TSH tests in these 125,840 or 1,031,888 patients ranged euro 12,584,000 to 103,188,800. Another study (99) analyzed the cost of resources consumed by frequent L-T4 dose changes over 24 months (laboratory testing, thyroid medications, general physician and specialist office visits, and emergency department visits/hospitalizations) in two groups of 227 hypothyroids each. Compared with the no-dose-adjustment patients (controls), significantly more patients needing dose adjustments (75 *vs.* 86%), were taking IM causing L-T4 malabsorption (PPIs, H2-receptor antagonists, calcium or iron supplements) (99). Overall resource utilization was higher in the ≥1-dose-adjustment group ($5,824/patient) *vs.* controls (USD 3,166/patient), peaking in the ≥3-dose-adjustment subgroup ($8,220/patient). The ≥1-dose-adjustment group experienced a 40.3% increase in lost productivity *vs.* controls ($1,381 *vs.* $984), with a peak in the ≥3-dose adjustment subgroup ($1,833). Compared to controls, patients requiring adjustments had significantly higher TSH (5.07 ± 11.04 *vs.* 2.57 ± 2.51 U/ml), and more frequent TSH tests (84 *vs.* 73%) (99).

## Potential Future Developments

In the “Areas for future research” heading of the 2012 ATA guidelines (4), a section was devoted to “*Agents and conditions having an impact on L-thyroxine therapy and interpretation of thyroid tests*”. Except for reminding that the residual functioning thyroid tissue is a major factor for a given IM to cause thyroid dysfuntion and L-T4 dose adjustments, no ideas were presented (4).

Future developments have already occurred considering the availability of L-T4 formulations (liquid, softgel) that are refractory or much more resistant to IM than tablet L-T4 (9, 14, 98, 100, 109–114). In the author’s opinion, their use seems preferable to the strategies of (i) increasing stepwise the dose of L-T4 (with associated frequent monitoring of TFTs and risk of iatrogenic thyrotoxicosis if the IM is decreased in dose or withdrawn); (ii) adding supplementation with either 1 g/d (115) or 0.5 g/d (116) vitamin C to acidify the intragastric pH. In the Argentinian study, the 28 patients had no known cause for UTH (115), while in the Colombian study, the 31 patients had endoscopy/gastritis-proven gastritis (116). Both 2-month-long trials with vitamin C (115, 116) lack formal pharmacokinetics studies and challenge of patients with IM-associated UTH. Indeed, coadministration of acidic beverages is one way of solving the problem of decreased bioavailability of drugs whose bioavailability under conditions of increased intragastric pH (117). Further to T4, there are a number of other drugs with decreased absorption at high intragastric pH, such as ketoconazole, itraconazole, atazanavir, cefpodoxime, enoxacin, dipyridamole, raltegravir, alendronate, digoxin, and nifedipine, to name a few (117). The other way is the development of “*formulations that can minimize or mitigate the effects of increased gastric pH on the bioavailability*” (117).

Considering the magnitude of polypharmacy, particularly in the elderly (118), and the aforesaid unfavorable impact of UTH on metabolic and CVD outcomes, more research is needed to substantiate those outcomes in the setting of IM-associated UTH. Once pejorative outcomes are confimed, monitoring of hypothyroid patients under IM should be tightened, with the biochemical monitoring not restricted to TSH solely.

## Author Contributions

SB made the work, drafted the article, revised it, and gave the final approval for the publication.

## Conflict of Interest

IBSA Institut Biochimique SA (Lugano, Switzerland) and IBSA Farmaceutici Italia s.r.l. furnished the principal investigator (SB) with novel formulations to conduct studies cited in the reference list. However, IBSA had no role in any phase of the writing of the above studies and this manuscript. Furthermore, SB was an invited speaker at symposia organized by IBSA.
